# Exercise Reduces Morphine-Induced Hyperalgesia and Antinociceptive Tolerance

**DOI:** 10.1155/2021/6667474

**Published:** 2021-09-27

**Authors:** Xingrui Gong, Rongmei Fan, Qinghong Zhu, Xihong Ye, Yongmei Chen, Mazhong Zhang

**Affiliations:** ^1^Department of Anesthesiology, Xiangyang Central Hospital, Affiliated Hospital of Hubei University of Arts and Science, Xiangyang, Hubei, China; ^2^Department of Anesthesiology and Pediatric Clinical Pharmacology Laboratory, Shanghai Children's Medical Center, Shanghai Jiao Tong University School of Medicine, Shanghai, China; ^3^Department of Respiratory, Shiyan Renmin Hospital (Affiliated Hospital of Hubei University of Medicine), Shiyan, Hubei, China; ^4^Department of Laboratory, Shiyan Taihe Hospital (Affiliated Hospital of Hubei University of Medicine), Shiyan, Hubei, China; ^5^Department of Laboratory, Xiangyang Central Hospital, Affiliated Hospital of Hubei University of Arts and Science, Xiangyang, Hubei, China

## Abstract

Chronic morphine intake for treating various pain is frequently concomitant with morphine-induced hyperalgesia and tolerance. The mechanisms can be explained by the activation of p38-MAPK proteins in microglia in the spinal cord horn. Exercise has been shown to prevent the development of microglia overactivation. Thus, we designed to test whether exercise prevents the morphine-induced hyperalgesia and tolerance as well as suppression of p38 phosphorylation. A p38 inhibitor SB203580, exercise, and exercise preconditioning were used for treating morphine-induced hyperalgesia and tolerance development in the present study. The behavior tests for hyperalgesia and tolerance were performed in male Wistar rats before and after morphine administration. Western blotting and immunostaining for examining phosphorylated-p38 expression were performed after the behavior tests. Our results showed that SB203580 and exercise, but not exercise preconditioning, prevented the occurrence of morphine-induced hyperalgesia and tolerance. Meanwhile, exercise decreased morphine-induced phosphorylated-p38 overexpression. In summary, exercise prevented the development of morphine-induced hyperalgesia and tolerance. The mechanism may be related to inhibition of p38 phosphorylation.

## 1. Introduction

Morphine is a classical drug and widely used for acute and chronic pain. Chronic morphine therapy induces hyperalgesia and antinociceptive tolerance [1]. Hyperalgesia is a sensitized condition that opioids paradoxically cause hypersensitivity to nociceptive stimulation, whereas tolerance is characterized by a progressive lack of antinociceptive effect of opioids which can be overcome by increasing the dosage. Previous studies have demonstrated that p38 phosphorylation plays a key role in the formation of hyperalgesia and tolerance by releasing a multitude of inflammatory mediators from microglia, including IL-1*β*, IL-6, and TNF-*α* [2, 3]. Inflammatory mediators sensitize spinal dorsal horn neuron and generate hypersensitive to pain stimulation. In addition, morphine-induced hyperalgesia has been reported to depend on activation of microglia via P2X4 receptor and through internal protein p38 phosphorylation, which leads to opioid tolerance and decreased pain threshold for nociceptive stimulation [2]. Generally, microglia activation contributes to morphine-induced hyperalgesia and antinociceptive tolerance. Thus, targeting p38 in microglia may be an effective approach to reduce morphine-induced hyperalgesia and tolerance.

Exercise and exercise preconditioning have been reported to alleviate inflammation, prevent microglia activation, inhibit macrophage infiltration, and reduce numbers of proinflammatory monocytes [4–6]. Further, exercise has been considered as a nonpharmacological method of reducing adverse effect of long-term opioid administration, and exercise increases pain threshold through stimulating endogenous opioid release in neuropathic pain models [7]. Consequently, exercise may reverse morphine-induced hyperalgesia and antinociceptive tolerance. We tested p-p38 inhibitor SB203580 on morphine-induced hyperalgesia and tolerance and observed exercise in reducing morphine-induced hyperalgesia and tolerance, as well as p-p38 expression in spinal dorsal horn.

## 2. Methods

### 2.1. Animals and Reagent Preparation

The experiments were conducted in accordance with the animal care and use committee of Shanghai Children's Medical Center, affiliated to the Shanghai Jiao Tong University School of Medicine, and under the guideline of the National Institutes of Health. Adult male Wistar rats (200-220 g) were bought from Shanghai Experimental Animal Center. The rats were housed 3 per cage and had *ad libitum* access to food and water with 12-hour light/dark circle. The temperature and humidity were controlled at 23°C and 60%, respectively. Animals were randomly allocated to each group (*n* = 6 per group). The drug was intrathecally (IT) injected under inhalational anesthesia with 3% isoflurane by one researcher, and thermal latency was measured by the other researcher who was blinded to the group allocation [8]. Morphine hydrochloride, bought from Shengyang First Pharmaceutical Factory, was diluted with normal saline, and SB203580 was purchased from Calbiochem (La Jolla, CA, USA), which was dissolved in 10% DMSO with saline.

### 2.2. Morphine Injection and Behavior Tests

Morphine, 30 mg/kg, was injected subcutaneously once daily for 7 days to induce hyperalgesia and antinociceptive tolerance according to a previous study [9]. The drug was injected at 10 AM subcutaneously, and the behavior tests of hot plate and tail-flick latency were performed at 9 AM and 30 minutes later after morphine administration.

Rats received isoflurane general anesthesia with 5% induction and 3% maintenance using an inhalational anesthesia system (Penlon Sigma Delta, Louisville, KY, USA). After anesthesia, the rat's back hair was shaved and a 50 *μ*L microinjector was used for administration of the SB203580. As soon as a tail flick was observed, 10 *μ*L SB203580 was injected via L5-6 spine. After the injection, anesthesia was terminated and the rat was returned to its cage for behavior tests.

For the hot-plate test, paw withdrawal latency was measured by an apparatus (Shandong Academy of medical Sciences Co, China), whose temperature was controlled at 54 ± 0.2°C. The researcher observed the rat's behavior through a transparent cage, surrounding the heat plate. As the rat was put into the cage, the timer was started, and if licking or flicking of hind paw or jumping behavior was observed, the time between the onset of thermal stimulation and occurrence of withdraw behavior was recorded as the latency. Cut-off time was set 30 seconds in case of tissue injury.

For the tail-flick test, a thermostatic water bath (Shanghai Sanshen Medical Co, China) was used to generate a nociceptive stimulation, which was set up to a constant water temperature of 54 ± 0.2°C. Rat was restraint in a tubular, and its tail was immersed in water with a length of 4.5 cm. The latency was recorded as time needed to elicit a tail flick completely above the water. Cut-off was set 30 seconds to minimize the tissue injury.

### 2.3. Exercise Protocol and Exercise Preconditioning Protocol

Exercise protocol followed our previous study [10]: the rat was first placed in the running wheel and acclimated for 10 min; then, the wheel was started at a speed of 5 meters per minute for 5 min to warm up. The speed was then slowly increased to 20 meters per minute and maintained for 30 minutes. Exercise preconditioning protocol: rats received the exercise protocol for 2 weeks and ceased before morphine experiments. Exercise was performed at 5 PM.

### 2.4. Western Blotting

After the last behavior tests, rats were deeply anesthetized with 5% isoflurane and decapitated and lumbar spinal cords were obtained for Western blotting. The spinal dorsal horn of lumber enlargement was homogenized and centrifuged. The supernatant was collected and quantified by bicinchoninic acid assay. Then, the sample was separated by SDS-PAGE 10% and transferred to nitrocellulose filter membrane and incubated with primary antibodies with anti-p-p38 (1 : 800, rabbit, cell signaling tech, USA) and anti-GAPDH (1 : 6000, mouse, protein tech, USA) concomitantly overnight at 4°C. Then, the membrane was incubated with secondary antibodies with fluorescence conjugate goat anti-rabbit and anti-mouse (1 : 10000, cell signaling tech, USA) and visualized by Odyssey infrared system. The ratio of p-p38/GAPDH relative to sedentary control was calculated for statistically analysis using the Image J software.

### 2.5. Immunostaining

After the last behavior tests, rats were deeply anesthetized and decapitated. The spinal cords were collected and fixed with 4% buffered paraformaldehyde at 4°C overnight, followed by 15% and 30% sucrose for dehydration. The spinal cord was then cut into 10 *μ*m thickness by a cryostat and incubated with an anti-p-p38 primary antibody (1 : 100, rabbit; Cell Signaling, USA), followed by the Alexa 555-conjugated sheep anti-rabbit secondary antibody (1 : 100; Invitrogen, USA) for visualization. Observation was done using a microscope (DM6000B, Leica, Germany).

### 2.6. Statistical Analysis

The data was presented by mean ± SEM. Statistical analysis used unpaired *t*-tests or one- or two-way ANOVA with repeated measures, and if a statistically significant difference was observed, Bonferroni post hoc analysis was followed. Dataset with nonnormal distribution was analyzed by nonparametric tests (Mann-Whitney and Kruskal-Wallis as indicated). Values of *P* < 0.05 were considered statistically significant. Statistical analysis was processed by using Prism (version 5.01, GraphPad software Co, CA).

## 3. Results

### 3.1. Pharmacological Blockage of p38 Reduces Morphine-Induced Hyperalgesia and Tolerance

**Figure 1:**
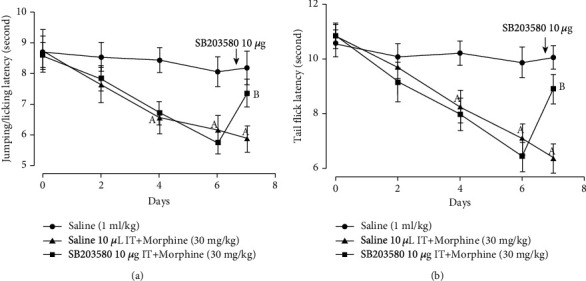
IT injection of SB203580 10 *μ*g prevented morphine-induced hyperalgesia in the (a) hot-plate test and (b) tail-flick test. Morphine 30 mg/kg was injected daily, and behavior tests were performed 1 h before morphine subcutaneous injection. On 7^th^ day, SB203580 10 *μ*g was IT injected and behavior tests were performed. IT: intrathecal. “a” denotes the statistically significant difference compared to the saline group, and “b” denotes the statistically significant difference compared to the saline+morphine group.

The results showed that morphine-induced hyperalgesia was established after 3 days of administration. Pain threshold for the hot-plate test (*F* = 16.7, *P* = 0.01; 4^th^: *t* = 3.10, *P* = 0.01; 6^th^: *t* = 3.80, *P* = 0.01, *n* = 6, two-way ANOVA with repeated measures followed by Bonferroni post hoc analysis, Figure 1(a)) and tail-flick test (*F* = 45.5, *P* = 0.01; 4^th^: *t* = 3.00, *P* = 0.01; 6^th^: *t* = 4.60, *P* = 0.01, *n* = 6, two-way ANOVA with repeated measures followed by Bonferroni post hoc analysis, Figure 1(b)) was different from the saline group in the experiment, while IT injection of SB203580 10 *μ*g reversed morphine-induced hyperalgesia in the hot plate (*F* = 5.70, *P* = 0.01; *t* = 3.03, *P* = 0.04, *n* = 6, one-way ANOVA followed by Bonferroni post hoc analysis, Figure 1(a)) and tail-flick test (*F* = 14.3, *P* = 0.01; *t* = 5.11, *P* = 0.01, *n* = 6, one-way ANOVA followed by Bonferroni post hoc analysis, Figure 1(b)).

**Figure 2:**
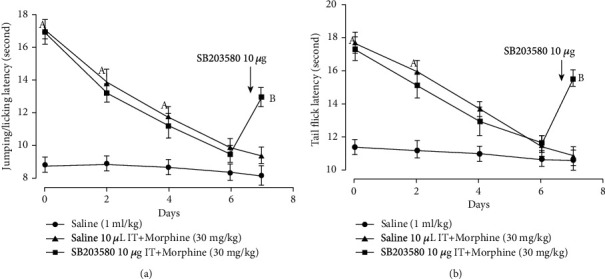
IT injection of SB203580 10 *μ*g prevented morphine tolerance in the (a) hot-plate test and (b) tail-flick test. Morphine 30 mg/kg was injected daily, and behavior tests were performed 30 minutes after morphine subcutaneous injection. On 7^th^ day, SB203580 10 *μ*g was IT injected before behavior tests. “a” denotes the statistically significant difference compared to the saline group, and “b” denotes the statistically significant difference compared to the saline+morphine group.

Morphine subcutaneous injection increased pain threshold in the hot-plate test (*F* = 37.5, *P* = 0.01; 0^day^: *t* = 9.68, *P* = 0.01; 2^ed^: *t* = 5.15, *P* = 0.01; 4^th^: *t* = 3.00, *P* = 0.03, two-way ANOVA with repeated measures followed by Bonferroni post hoc analysis, *n* = 6, Figure 2(a)) and tail-flick test (*F* = 33.6, *P* = 0.01; 0^day^: *t* = 6.96, *P* = 0.01; 2^ed^: *t* = 4.59, *P* = 0.01, two-way ANOVA with repeated measures followed by Bonferroni post hoc analysis, *n* = 6, Figure 2(b)). Morphine antinociceptive effect at the 6^th^ day in the hot-plate test (*t* = 1.29, *P* = 0.30, Figure 2(a)) and the tail-flick test (*t* = 1.18, *P* = 0.32, Figure 2(b)) was not different from the saline group. On 7^th^ day, IT injection of SB203580 10 *μ*g reduced morphine antinociceptive tolerance in the hot plate (*F* = 20.3, *P* = 0.01; *t* = 6.46, *P* = 0.01, one-way ANOVA followed by Bonferroni post hoc analysis, Figure 2(a)) and tail-flick tests (*F* = 24.1, *P* = 0.01; *t* = 8.26, *P* = 0.01, one-way ANOVA followed by Bonferroni post hoc analysis, Figure 2(b)).

### 3.2. Exercise Reduces Morphine-Induced Hyperalgesia and Tolerance

**Figure 3:**
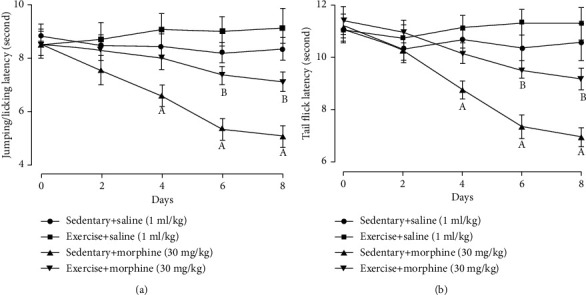
Exercise training prevented the development of morphine-induced hyperalgesia in the (a) hot-plate test and (b) tail-flick test. Rats received exercise training for 30 minutes every afternoon. Morphine 30 mg/kg was injected daily, and behavior tests were performed 1 h before morphine subcutaneous injection. “a” denotes the statistically significant difference compared to the sedentary+saline group, and “b” denotes the statistically significant difference compared to the sedentary+morphine group.

After 3 days of morphine administration, morphine-induced hyperalgesia in the hot-plate test (*F* = 4.66, *P* = 0.01; 4^th^: *t* = 3.16, *P* = 0.01; 6^th^: *t* = 5.24, *P* = 0.01; 8^th^: *t* = 5.58, *P* = 0.01, two-way ANOVA with repeated measures followed by Bonferroni post hoc analysis, *n* = 6, Figure 3(a)) and tail-flick test (*F* = 4.43, *P* = 0.02; 4^th^: *t* = 3.98, *P* = 0.01; 6^th^: *t* = 4.41, *P* = 0.01; 8^th^: *t* = 4.61, *P* = 0.01, two-way ANOVA with repeated measures followed by Bonferroni post hoc analysis, *n* = 6, Figure 3(b)) was established. Exercise inhibited the induction of hyperalgesia in the hot-plate test (6^th^*t* = 3.86, *P* = 0.01; 8^th^*t* = 3.76, *P* = 0.01, Figure 3(a)) and tail-flick test (6^th^*t* = 3.92, *P* = 0.01; 8^th^*t* = 4.02, *P* = 0.01, Figure 3(b)) after chronic morphine injection. Exercise did not affect pain threshold in the hot-plate test and tail-flick test in the control rats received normal saline.

**Figure 4:**
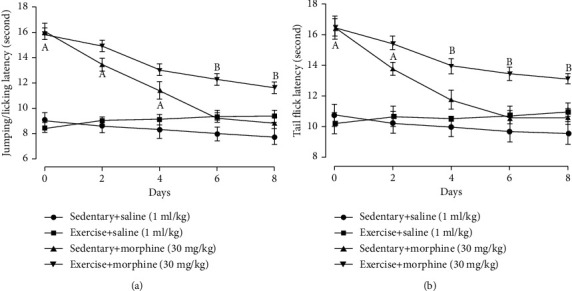
Exercise training prevented the development of morphine antinociceptive tolerance in the (a) hot-plate test and (b) tail-flick test. Rats received exercise training for 30 minutes every afternoon. Morphine 30 mg/kg was injected daily, and behavior tests were performed 30 minutes after morphine subcutaneous injection. “a” denotes the statistically significant difference compared to the sedentary+saline group, and “b” denotes the statistically significant difference compared to the sedentary+morphine group.

Morphine administration increased the pain threshold in the hot-plate test (*F* = 27.1, *P* = 0.01; 0^day^: *t* = 9.66, *P* = 0.01; 2^ed^: *t* = 6.70, *P* = 0.01; 4^th^: *t* = 4.25, *P* = 0.01, two-way ANOVA with repeated measures followed by Bonferroni post hoc analysis, *n* = 6, Figure 4(a)) and tail-flick test (*F* = 13.0, *P* = 0.01; 0^day^: *t* = 6.89, *P* = 0.01; 2^ed^: *t* = 4.21, *P* = 0.01, two-way ANOVA with repeated measures followed by Bonferroni post hoc analysis, *n* = 6, Figure 4(b)). After four days of morphine administration, morphine antinociceptive effect was not different from the saline group in the hot-plate test (6^th^: *t* = 1.87, *P* = 0.09; 8^th^: *t* = 1.48, *P* = 0.17, Figure 4(a)) and tail-flick test (4^th^: *t* = 1.95, *P* = 0.08; 6^th^: *t* = 1.11, *P* = 0.29; 8^th^: *t* = 1.37, *P* = 0.20, Figure 4(b)). Exercise reduced the antinociceptive tolerance in the hot-plate test (6^th^: *t* = 5.31, *P* = 0.01; 8^th^: *t* = 4.51, *P* = 0.01, Figure 4(a)) and tail-flick test (4^th^: *t* = 2.93, *P* = 0.02; 6^th^: *t* = 4.74, *P* = 0.01; 8^th^: *t* = 3.94, *P* = 0.01, Figure 4(b)) after morphine administration.

### 3.3. Exercise Reduces Morphine-Induced Hyperalgesia and Tolerance via Inhibition of p-p38 Expression

**Figure 5:**
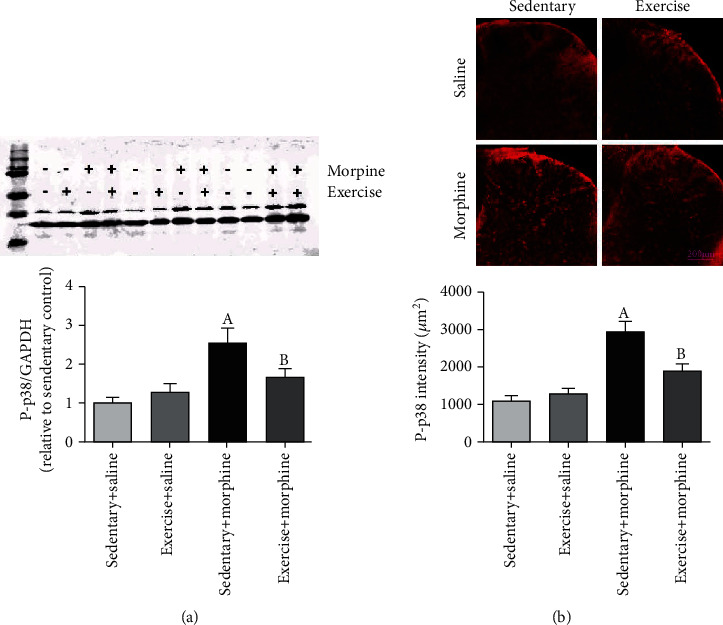
Morphine increased p-p38 expression in the spinal dorsal horn, and exercise training decreased that effect by (a) Western blot and (b) immunostaining image of p-p38 in the spinal dorsal horn. “a” denotes the statistically significant difference compared to the sedentary+saline group, and “b” denotes the statistically significant difference compared to the sedentary+morphine group.

Western blotting results showed that an 8-day morphine administration increased p-p38 expression (*F* = 7.86, *P* = 0.01; *t* = 5.34, *P* = 0.01, one-way ANOVA followed by Bonferroni post hoc analysis, *n* = 6, Figure 5(a)), and exercise decreased morphine-induced p-p38 overexpression (*t* = 3.83, *P* = 0.04). Exercise did not affect p-p38 expression of rats received normal saline (*t* = 0.92, *P* = 0.38). Representative immunofluorescence image of p-p38 in the spinal dorsal horn is shown in Figure 5(b). An 8-day morphine administration increased p-p38 expression intensity (*F* = 16.5, *P* = 0.01; *t* = 6.44, *P* = 0.01, one-way ANOVA followed by Bonferroni post hoc analysis, *n* = 4, Figure 5(b)), and exercise decreased morphine-induced p-p38 overexpression (*t* = 3.57, *P* = 0.03). Exercise did not affect p-p38 expression of rats received normal saline (*t* = 0.8, *P* = 0.36).

### 3.4. Exercise Preconditioning Did Not Affect the Development of Morphine-Induced Hyperalgesia and Tolerance

**Figure 6:**
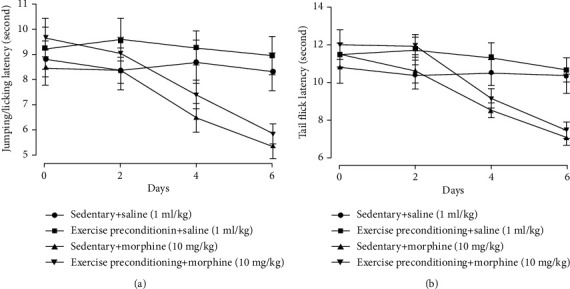
Exercise preconditioning did not prevent the development of morphine-induced hyperalgesia in the (a) hot-plate test and (b) tail-flick test. Rats first received 30-minute exercise training for 2 weeks, then morphine 30 mg/kg was injected daily, and behavior tests were performed 1 h before morphine subcutaneous injection.

**Figure 7:**
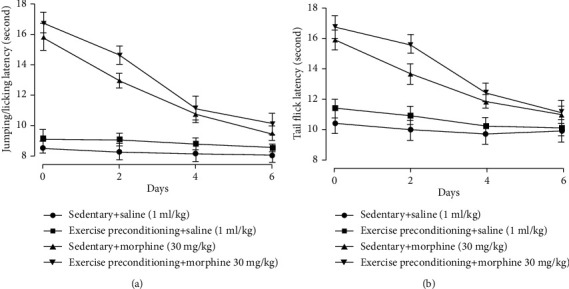
Exercise preconditioning did not prevent the development of morphine antinociceptive tolerance in the (a) hot-plate test and (b) tail-flick test. Rats first received 30-minute exercise training for 2 weeks, then morphine 30 mg/kg was injected daily, and behavior tests were performed 30 minutes after morphine subcutaneous injection.

After 2 weeks of exercise preconditioning, the baseline value of latency to hot plate (*t* = 0.37, *P* = 0.72, one-way ANOVA, *n* = 6, Figure 6(a)) and tail flick (*t* = 0.64, *P* = 0.54, one-way ANOVA, *n* = 6, Figure 6(b)) was not different from sedentary groups. After 6 days of morphine injection, morphine-induced hyperalgesia and tolerance were established in the hot plate and tail-flick tests. However, exercise preconditioning did not affect the development of morphine-induced hyperalgesia in the hot-plate test (*F* = 1.04, *P* = 0.33, two-way ANOVA, *n* = 6, Figure 6(a)) and tail-flick test (*F* = 1.07, *P* = 0.32, two-way ANOVA, *n* = 6, Figure 6(b)). In addition, exercise preconditioning did not affect morphine antinociceptive tolerance in the hot-plate test (*F* = 3.07, *P* = 0.11, two-way ANOVA, *n* = 6, Figure 7(a)) and tail-flick test (*F* = 2.77, *P* = 0.13, two-way ANOVA, *n* = 6, Figure 7(b)).

## 4. Discussion

In the study, our results showed that IT injection of p38 inhibitor SB203580 reversed morphine-induced hyperalgesia and antinociceptive tolerance. In parallel, exercise prevented the development of morphine-induced hyperalgesia and tolerance, and exercise decreased morphine-induced p-p38 overexpression. However, exercise preconditioning failed to inhibit morphine-induced hyperalgesia and tolerance. Our results proved that exercise is an effective method for management of morphine-induced hyperalgesia and antinociceptive tolerance.

In our research, we established animal models of morphine-induced hyperalgesia and morphine antinociceptive tolerance by injecting morphine once daily for 7 consecutive days. These were shown by decreased pain threshold in the hot-plate test and the tail-flick test, as well as reduced thermal latency to the morphine. In addition, our results showed that morphine continuous injection increased p-p38 expression in the spinal cord. Previous study has demonstrated that p-p38 is increased after chronic morphine intake, and p-p38 is mainly located in microglia [11]. p38 phosphorylation activates downstream signals and generates a host of inflammatory mediators, including IL-1*β*, IL-6, and TNF-*α*. The inflammatory mediators sensitize spinal dorsal horn neuron and generate hypersensitive to pain, which leads to morphine-induced hyperalgesia and tolerance [12, 13]. In our study, we used p38 inhibitor, SB203580, to treat the hyperalgesia and tolerance induced by chronic morphine intake; the results showed that pharmacologically blocked p38 in the spinal cord reversed the established morphine-induced hyperalgesia and tolerance. Our results confirmed that p38 phosphorylation, at least in part, contributed to the development of morphine-induced hyperalgesia and tolerance.

Previous study has demonstrated that morphine prolongs chronic pain by enhancing the proinflammatory response in the spinal cord [14]. In fact, a lot of studies have focused on morphine in chronic pain management and demonstrated that morphine is not an ideal drug for clinical treating chronic pain due to tolerance, hyperalgesia, addiction, and respiratory depression. Surprisingly, chronic morphine alone is sufficient to induce hyperalgesia to noxious or nonnoxious stimulation by a mechanism of enhanced microglia activation [2]. Overactivated microglia release BDNF and result in downregulation of the K^+^-Cl^−^ cotransporter KCC2, impairing Cl^−^ homeostasis in rat spinal lamina l neurons. Microglia-mediated disruption of neuronal Cl^−^ homeostasis leads to increased excitability of the lamina l projection neurons and contributes to the enhanced sensitivity to noxious or nonnoxious stimulus. As a result, morphine alone induced hyperalgesia and the behavior response to stimulation in our experiments confirmed that.

Further, we found that exercise training reduced chronic morphine-induced hyperalgesia and antinociceptive tolerance which was verified by decreased thermal latency in hot-plate and tail-flick tests. Exercise has been reported to have antinociceptive effect and inhibit central nervous inflammation [4, 15]. In the central nervous system, microglia are the main source of inflammation; they change their shape and generate inflammatory cytokines in response to stimulation [16]. In the study, we found that exercise decreased p-p38 overexpression in the spinal dorsal horn. p38 is a key protein located in microglia and generates “inflammatory soup” which leads to hyperalgesia and tolerance. The other study has demonstrated that exercise provides an analgesic effect via increasing endogenous opioid release [7], but whether the increased endogenous opioids also have tolerance or hyperalgesia remains to be further studied. However, our results showed exercise suppressed morphine-induced hyperalgesia and tolerance by suppression of p-p38 overexpression. Our study provides a new approach to reduce the hyperalgesia and tolerance for patients who need a chronic opioid treatment.

Although exercise suppressed microglia and internal p38 phosphorylation, the mechanisms may be also attributed to other reasons. Recent studies have shown that exercise induces anti-inflammatory cytokine release from skeletal muscle and switches the classically activated macrophage to alternatively activated macrophage in the skeletal muscles, which release anti-inflammatory cytokine IL-10 [17]. Generally, exercise increases circulating numbers of anti-inflammatory monocyte in the periphery [18]. The blood-derived monocytes or macrophages infiltrate into the spinal cord and play an anti-inflammatory role by releasing IL-10, which stimulates proinflammatory microglia and switches to alternatively activated microglia [19]. The alternatively activated microglia release IL-10 and IL-4 and suppress proinflammatory response, which contributes to morphine-induced hyperalgesia and tolerance [20]. Thus, exercise shows anti-inflammatory effect both in the central and peripheral system.

On the contrary, our results found that exercise preconditioning did not affect morphine-induced hyperalgesia and tolerance, which was probably due to that exercise-induced microglia suppression was disappeared quickly as the exercise ceased. When the morphine was continuously delivered, the effect of exercise preconditioning decreased quickly and failed to inhibit later microglia activation; thus, morphine-induced hyperalgesia and tolerance were established similar as sedentary rats. Consequently, exercise, rather than exercise preconditioning, is effective in the prevention of the development of morphine-induced hyperalgesia and antinociceptive tolerance.

In all, we found that exercise reduced chronic morphine-induced hyperalgesia and antinociceptive tolerance as well as suppression of p-p38 overexpression in the spinal dorsal horn. Our research provides a new approach to the management of chronic opioid-induced hyperalgesia and antinociceptive tolerance.

## Data Availability

Data is available upon request.
